# Toward the use of novel alternative methods in epilepsy modeling and drug discovery

**DOI:** 10.3389/fneur.2023.1213969

**Published:** 2023-08-31

**Authors:** Claudia Miguel Sanz, Miriam Martinez Navarro, Daniel Caballero Diaz, Gentzane Sanchez-Elexpuru, Vincenzo Di Donato

**Affiliations:** ZeClinics SL, IGTP (Germans Trias I Pujol Research Institute), Badalona, Spain

**Keywords:** epilepsy, genetic models, Dravet syndrome, zebrafish, alternative methods, anti-epileptic drug screening, 3Rs

## Abstract

Epilepsy is a chronic brain disease and, considering the amount of people affected of all ages worldwide, one of the most common neurological disorders. Over 20 novel antiseizure medications (ASMs) have been released since 1993, yet despite substantial advancements in our understanding of the molecular mechanisms behind epileptogenesis, over one-third of patients continue to be resistant to available therapies. This is partially explained by the fact that the majority of existing medicines only address seizure suppression rather than underlying processes. Understanding the origin of this neurological illness requires conducting human neurological and genetic studies. However, the limitation of sample sizes, ethical concerns, and the requirement for appropriate controls (many patients have already had anti-epileptic medication exposure) in human clinical trials underscore the requirement for supplemental models. So far, mammalian models of epilepsy have helped to shed light on the underlying causes of the condition, but the high costs related to breeding of the animals, low throughput, and regulatory restrictions on their research limit their usefulness in drug screening. Here, we present an overview of the state of art in epilepsy modeling describing gold standard animal models used up to date and review the possible alternatives for this research field. Our focus will be mainly on *ex vivo*, *in vitro*, and *in vivo* larval zebrafish models contributing to the 3R in epilepsy modeling and drug screening. We provide a description of pharmacological and genetic methods currently available but also on the possibilities offered by the continued development in gene editing methodologies, especially CRISPR/Cas9-based, for high-throughput disease modeling and anti-epileptic drugs testing.

## 1. Introduction to causes of epilepsy and available treatments

Epilepsy, one of the most common neurological disorders, affects around 50 million people according to the World Health Organization (WHO). It is a severe neurological disorder characterized by recurrent seizures (1). A seizure is defined as “a transient occurrence of signs and/or symptoms due to abnormal excessive or synchronous neuronal activity in the brain” (2). In 2017, International League Against Epilepsy (ILAE) approved and published an updated classification of seizure types (3, 4). This classification was generated for practical use in the clinical setting, but it can also be used by researchers with specific purposes. Depending on their onset, seizures can be classified into focal (originated in localized parts of the brain), general (originated from extensive regions in both hemispheres of the brain) and unknown. Focal seizures can be further classified based on the level of awareness, understood as the person’s awareness of self and environment during the seizure. In addition, both focal and generalized seizures can be divided into motor (e.g., tonic or clonic) and non-motor (e.g., sensorial signs as absence) seizures, and subdivided into different categories described in detail by Devinsky et al. (5) and Fisher et al. (6).

Epilepsy is considered a spectrum disorder with highly diverse etiology, comprising structural, genetic, metabolic, autoimmune and infection-related causes. Structural causes (5, 7) refers to abnormal structural brain defects that are known to substantially increase the risk of seizures. These structural abnormalities can be congenital or acquired, like brain tumors, strokes or head trauma (8). The epileptic syndromes are defined by the ILAE as “a characteristic cluster of clinical and electroencephalographic (EEG) features, often supported by specific etiological findings.” The correct diagnosis of an epileptic syndrome is crucial since it usually has important implications in the prognosis and treatment (9).

### 1.1. Genetic basis of epileptic syndromes

Genetic causes of epilepsy usually involve single-gene mutations affecting ion channels, synaptic support proteins, mTOR pathway regulators chromatin remodeling and trascription regulators (10). These types of epilepsies are very diverse and in most cases the underlying genes have not been identified yet (8). Some of the identified single gene mutations causing epilepsy are in the SCN1A (11), SCN8A and HCN1 genes for Dravet Syndrome (DS) (12), in the GABRA1 gene (A322D mutation) for Juvenile myoclonic epilepsy (13), in the LIS1 gene for Classical Lissencephaly (14), in the STXBP1, DNM1, DEPDC5 and GRIN2B genes for Epileptic Encephalopathy (15), in the CHD2 (16) and GABRB3 (17) genes for Lennox–Gastaut syndrome and PCDH19 genes for PCDH19 female epilepsy (18). Over the last years, thanks to the constant improvements in sequencing technologies, a growing number of novel variants have been discovered by analyzing large cohorts of patients within the framework of several international collaborations. Among those, the Epi4k consortium, composed by more 60 researchers in USA, Australia and United Kingdom, aims to unravel, by sequencing and analyzing over 4,000 genomes, genetic causes of under studied forms of epilepsy (Infantile Spasms and Lennox–Gastaut Syndrome) and identify novel *de novo* or rare pathogenic variants (19, 20). A similar example of an inter-institutional effort is the Epi25 collaborative established in 2014 with the aim to perform exome sequencing of 25.000 epilepsy patients and correlate the data sequencing results with phenotypic data in order to reach a better patient stratification and genotype/phenotype spectrum correlation. The work of the collaborative led to a very recent publication releasing data from the largest analysis of copy number variants as risk factor for epilepsy performed to date, including discovery of novel variants and definition of phenotypic signatures for almost 20 clinical categories (21). On the same line, the International League Against Epilepsy (ILAE) Consortium on Complex Epilepsies run a genome-wide analysis of nearly 45,000 people which led to the identification of 16 genetic loci associated with generalized epilepsy (11 of which newly identified) and, within these loci, 21 genes coding for ion-channel subunits (SCN1A, SCN2A, SCN3A, GABRA2, KCNN2, KCNAB1, and GRIK1), transcription factors (ZEB2, STAT4, and BCL11A), synaptic transmission regulators (STX1B), etc. (22).

Despite the aforementioned efforts to untangle the complexity of the genetics underlying epileptic phenotypic heterogeneity is high. Indeed, *de novo* or familial mutations in epilepsy-related genes are characterized by a variable expressivity, thus an extremely variable phenotyping spectrum ranging from generalized epilepsies to severe encephalopathies (23, 24). The causes for these very diverse phenotypic outcomes linked to gene modification are hotly debated in the genetics community. Among other causes, can be found the probable involvement of modifier loci, somatic mosaicism, repeated expansion and significant environmental variables. Genetic modifiers, which interact with the primary mutation and modulate the disease severity, have been identified for the SCN1a gene in mice (25) and human patients (26). In other cases, post-zygotically acquired mutations can be accumulated in a tissue specific manner affecting subpopulations in a variable number of neuronal cells in different brain regions (27). For example, variants of the GLI3 gene in the germline give rise to Pallister Hall, a syndrome that includes congenital anomalies as Hypothalamic hamartoma (HH), while variants limited to (or enriched in) the hypothalamus can lead to isolated HH (28).

Other cases where genomic instability plays a role in the severity or age of onset of epileptic symptoms in human patients have caught the interest of clinicians and researchers in the field during the last years. Importantly, until the advent of the advanced sequencing technologies, the search for pathogenic variants was mainly focused in the coding regions of the identified genes and those could not justify the incomplete penetrance and variable expressivity of the pathogenesis nor the high percentages of individuals affected even not being carriers of the mutations. Indeed, genetic linkage analysis, where the use of several molecular markers is employed to identify the location of a disease-causing variant, have provided the groundbreaking discovery that non-coding regions of defined genetic loci contribute to the etiology of forms of epilepsy. In particular, repeat expansions in non-coding regions of different genetic loci cause autosomal dominant forms of Familial adult onset myoclonus epilepsy (FAME) (29, 30). Intriguingly, the length of the repeats, which shows generational instability, correlates with the age of onset and severity of the detected phenotypes (31).

If on one hand the genetic complexity of epilepsy is a burden for understanding the pathophysiology, on the other hand the novel discoveries on somatic mosaicism and repeated expansion open an opportunity for better patient stratification and enhance the possibilities of diagnostic detection of the disease.

### 1.2. Environmental factors and comorbidities

In addition to genetic causes environmental causes have been identified for pathogenesis (8). A common risk factor for seizures and acquired epilepsy are infections. Epilepsies with infectious etiology are the ones in which seizures are the main symptom as a direct consequence of an infection. Seizures can be the only symptom, or can represent one symptom among other dysfunctions of the central nervous system (32). Epilepsies can also be a result of a metabolic disorder, although in most of the cases they will also have a genetic basis, or can also be a consequence of an immune disorder. Moreover, there are still some epilepsies of unknown etiology (8).

Moreover, it is important to note the significant negative impact of comorbidities in epilepsy. Comorbidity was defined by Feinstein as “any distinct additional entity that has existed or may occur during the clinical course of a patient who has the index disease under study” (33). Patients with epilepsy are affected by several diseases such as depression, anxiety, dementia, migraine, heart disease, peptic ulcers, and arthritis up to eight times more than the general population (34). In addition, some conditions such as psychiatric, endocrine/metabolic, and respiratory disorders are associated with worse seizure outcomes in the long-term (35). Various models have been generated to account for the relation between comorbid disorders. These models are not mutually exclusive and even within a single person, the same comorbid disease may be linked to epilepsy for a variety of reasons. It is particularly interesting the role of genetics in epilepsy and its comorbidities. Genetic mutations can be a shared risk factor, like for example in the SCN1A gene, where mutations predispose individuals to the development of epilepsy, but also a gait disorder. Genetic factors can also act as modifiers, impacting the relation between cause and effect, like for example the higher risk of epilepsy in carriers of the APOE4 allele after traumatic brain injury. The contribution of comorbidities to mortality in epilepsy is quite significant, underlying the relevance of the study of the causal mechanisms (34).

### 1.3. Overview on anti-seizure medications

Currently there is no effective treatment for epilepsy and most of the drugs used in the treatment of epilepsy are directed to treat the symptoms or seizures rather than treating the underlying disease. Therefore, although historically they have been named anti-epileptic drugs (AEDs), the term anti-seizure medications (ASMs) is nowadays more widely accepted. By definition, ASMs prevent or suppress the generation, propagation, and severity of epileptic seizures. The majority of ASMs work by altering voltage-gated ion channels, enhancing gamma aminobutyric acid (GABA)-mediated inhibition, interacting with synaptic release machinery, blocking ionotropic glutamate receptors, or a combination of these mechanisms (36). Some patients achieve seizure control with the use of one medication, however in many cases a combination of multiple medications is necessary. There are other types of approaches for the treatment of epilepsies, including surgery, neuromodulation devices or diet (5). Even with the currently available ASMs and other types of therapies, about one third of the patients do not achieve seizure control. This is partly due to the drug resistance that many patients with different types of epilepsy develop. In addition to resistance mechanisms, a critical issue contributing to the slow pace of novel ASM discovery is reliability of evaluation of compound efficacy in human patients starting from data generated with rodent models or NAMs. Performing the ADME (administration, distribution, metabolism and excretion) profiling of a molecule and assessing its capacity to cross the blood brain barrier (BBB) are challenging tasks and the results might not accurately predict the outcomes in patients, also considering inter individual susceptibility and differential response based on age to compound administration (37). Regarding ADME in *in vivo* models, it has to be taken into account that rodents eliminate drugs at a quicker rate than humans, making the generation of dose–response efficacy curves complicated. Nevertheless, longitudinal studies with rat or mouse models with multi-injections regimes followed by blood serum concentration analysis allow to study the pharmacodynamics of the administered molecules (38). Thereafter, the evaluation of the concentration of the compound reaching the CNS is estimated with the brain–blood or brain-plasma ratio, a model that correlates the brain-targeting ability of therapeutics with the CNS pharmacokinetics (39). This tool is more straightforward than other time consuming and invasive techniques such as microdialysis and *in situ* brain perfusion (40). Indeed, over the last years, *in silico* predictions based on available *in vivo* and *in vitro* data and molecular descriptors of the compounds of interest have been optimized to infer the BBB permeability of neurotherapeutics (41, 42). In *in vitro* models ADME studies cannot be directly performed, however cost effective assays can be used as indicators of the ADME fate of compounds *in vivo*. Among other parameters, it is possible to calculate the physicochemical properties as lipophilicity, solubility as well as its metabolic fate via hepatic microsome stability and plasma stability assays (43). In addition to these, multiple cell culture models derived from a variety of species have been developed to mimic the BBB and study molecule transport through this structure (44). With regard to whole embryo non animal studies, as the ones performed with the zebrafish model, it is possible to extrapolate relevant Absorption, Metabolism and Excretion values since zebrafish can adsorb and metabolize toxicants in a similar manner to that of mammals. In this case, zebrafish embryos are treated with selected compounds by waterborne exposure and collected at different exposure times for LC-HRMS analysis (45). This method allows the evaluation of the stability and toxicokinetic profile of novel molecules. Also in the zebrafish model, the brain-to-plasma concentration can be calculated and, interestingly, it has been shown that there is a correlation between the partition coefficient (Kp, brain) values obtained from the zebrafish and mice, indicating that zebrafish can be an alternative to rodent models to predict drug penetration in humans (46).

Taking into account all the previous considerations, there is an essential need for a better understanding of the basic mechanisms of the processes leading to epilepsy, the biological mechanisms of pharmacoresistance and the development of disease-modifying therapies. To achieve these goals, well established models of epilepsy are the most important prerequisite.

## 2. Current state of art in epilepsy models

**Figure 1:**
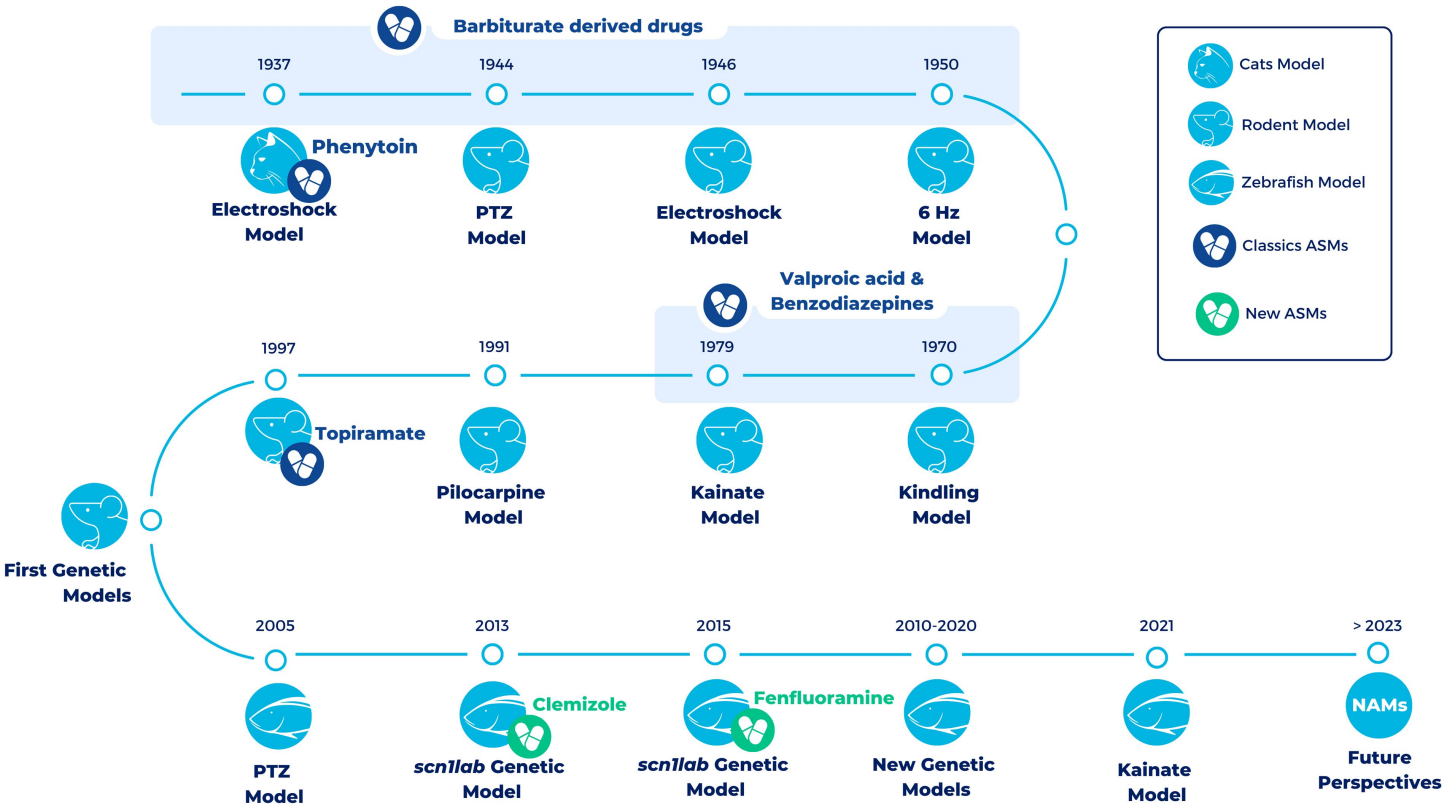
Timeline of the most representative vertebrate animal models in epilepsy over the last century. All models (exception of the electroshock models in cats) are still being used in the development of new treatments for epilepsy. During the first half of the twentieth century and up to the late 1990s, a large number of compounds with antiepileptic properties were discovered in these models (Classics ASMs). However, most of these compounds were discovered in pharmacoresistant models of epilepsy. The development of the first genetic models has allowed progress to be scored in the search for novel antiseizure medications that are able to overcome drug resistance. In the last decade, the use of zebrafish has led to the development of Fenfluramine (FDA-approved drug) and Clemizole (in DS clinical phases). In the future, it is hoped that new approach methodologies (NAMs), such as zebrafish, organoids and induced Pluripotent Stem Cells (iPSCs), will facilitate the discovery of new drugs useful for different types of epilepsy (new ASMs).

Over the years different animal models have been developed to study epilepsy (Figure 1). A very classic and widely used group of epilepsy models are the ones with an induction of seizures in wild-type animals. This induction can be electrical or chemical and in both cases it can be an acute or a chronic induction (47, 48).

Among the electrically induced acute seizures, the best-validated preclinical test is the maximal electroshock seizure (MES) test, in which an acute seizure is electrically induced in a normal non-epileptic animal. This test is very effective in identifying drugs against generalized tonic–clonic seizures (49). Another example of electrically induced acute seizures is the 6-Hz psychomotor seizure model of partial epilepsy, a model of pharmacoresistant epilepsy. This model, in which an electrical stimulation by low-frequency (6-Hz) is delivered through corneal electrodes, has been used both with mice and rats (50, 51). Repeated 6 Hz corneal stimulation in mice has also been used to successfully establish a kindling model showing resistance to ASMs (52). Kindling models are the models in which repeated non-convulsive stimuli are applied progressively producing a change in seizure response and finally reaching a fully kindled state with a stable seizure response to each stimulation (53). These models belong to electrically induced chronic seizures, and the best established model among them is the amygdala kindling rat model of temporal lobe epilepsy (TLE). In this model, there is a repeated application of electrical stimuli through a depth electrode in the basolateral amygdala of rats and this induces a permanent enhancement of seizure susceptibility together with other brain alterations that are similar to the ones occurring in human TLE. It was the first proposed model of pharmacoresistant partial epilepsy (48, 54).

On the other hand, there are chemically induced seizures. One of the most commonly used models of acute chemically induced seizures is the pentylenetetrazole (PTZ) test, which has been crucial for the identification of many ASMs that are clinically used today. PTZ is an antagonist of the type A receptor of γ-aminobutyric acid (GABAA). The administration of low doses of PTZ (sub-convulsive) in animal models can result in absence seizures (55), whereas higher doses (convulsive) produce generalized tonic–clonic seizures (56). PTZ has also been used to generate a chemically induced kindling model by the repeated administration of sub-convulsive doses (57). Although PTZ use is very extended in mice and rats, it is also routinely used in other models such as zebrafish (58).

Another important group of chronic models of epilepsy are models in which after inducing status epilepticus by chemical or electrical stimulation spontaneous recurrent seizures develop (48). Status epilepticus is defined by the ILAE as “a condition resulting either from the failure of the mechanisms responsible for seizure termination or from the initiation of mechanisms, which lead to abnormally prolonged seizures” (59). Although these models can be induced by electrical stimulus, the most extended models are the ones generated by either pilocarpine, cholinergic muscarinic agonist pilocarpine, or kainate, a cyclic analog of L-glutamate and an agonist of the ionotropic kainate receptors. Both pilocarpine and kainate represent post-status epilepticus models of TLE (48, 60).

The other main group of epilepsy models is the genetic animals models. With the description of more single gene mutations causing epilepsy, and the advancements in gene-editing techniques, more genetic animal models have been developed and validated (61). The generation of these models contributes to a better understanding of the mechanisms of epileptogenesis. A good example of this are the mouse models of lissencephalies (14, 62). In humans, heterozygous mutation or deletion of the lissencephaly gene (LIS1) leads to classical or type I Lissencephaly, causing cognitive deficits, severe seizures, and a serious disruption of cortical and hippocampal lamination. Before the generation of mouse models, how neurons communicate in Lis1-deficient brain was not well understood. The generation of a Type I Lissencephaly mouse model permitted the description of alterations in synaptic inhibition that may contribute to seizures and altered cognitive function, which can potentially lead to advances in novel therapeutic strategies (62). Moreover, the models of lissencephalies have been crucial for understanding the function of LIS1 and the pathways associated with it during brain development (14). Furthermore, genetic animal models have been fundamental for the advancement of therapeutic interventions. This is for example the case of the mouse models that have been generated for DS, which have also been extensively characterized (63–65). In one of these models, for example, treatment with low-dose clonazepam, a positive allosteric modulator of GABAA receptors, completely rescued the abnormal social behaviors and deficits in fear memory of these mice (66). In a more recent study, Hawkins et al. also demonstrated that treatment with soticlestat, a novel potent and highly selective brain-specific inhibitor of the CH24H enzyme, significantly improved Dravet-like phenotypes of Scn1a Dravet mouse models (67). In summary, the development of genetic animal models has been of relevance not only to expand the knowledge of the mechanisms of epileptogenesis, but also to move forward in the discovery of new potential therapies.

Despite the large number of models that have been established for the development of new therapies in epilepsy, 30% of the patients do not response to classic ASMs and consequently more research and new models are needed. The discovery of new ASMs requires the screening of large number of compounds and, therefore, the models need to be not only predictive of clinical activity, but also easy to perform and time and cost efficient.

## 3. Alternative models: toward the 3R in epilepsy model generation and drug screening

The high impact of epilepsy on patients and their communities highlights the urgent need to improve the understanding of its pathophysiology and develop efficient treatments for seizure regulation. However, the use of conventional *in vivo* and *in vitro* models based on rodents display substantial limitations and ethical concerns. Although rodent models may be particularly useful for predicting treatment responses in humans due to the greater similarities between the nervous systems of different mammalian species, variation in the genetic background of rodent strains can also result in opposing or contradictory results. Rodent models are also more expensive and require complicated, invasive procedures to study the role of genes in seizure mechanisms (68). Additionally, growing awareness of the sentience of animals and their experience of pain has led to the adoption of the 3Rs principle (replace, reduce, and refine) by all the ethical committees and whenever possible, novel alternative models to animal experimentation are recommended (69).

**Figure 2:**
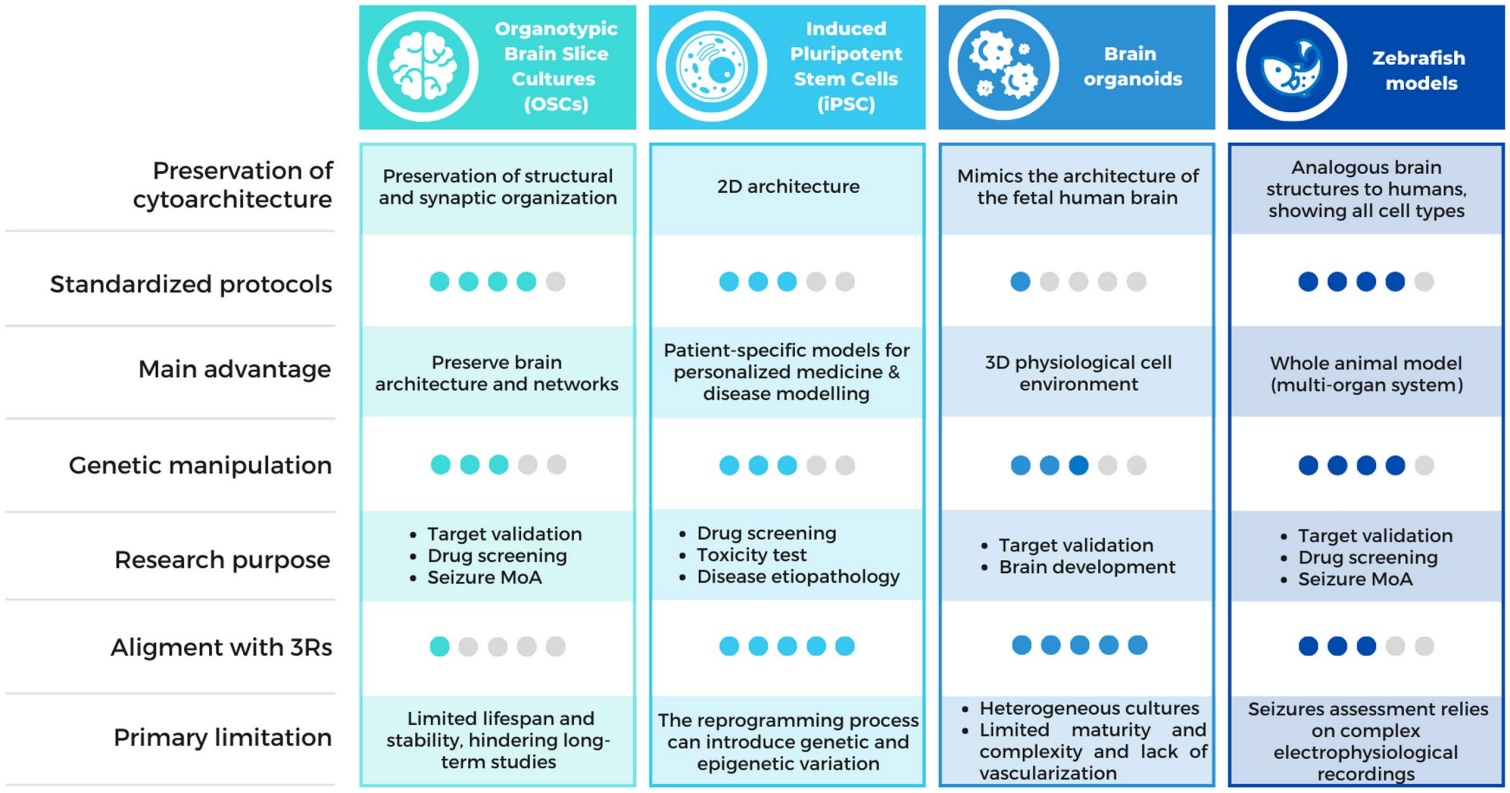
Comparison chart of the alternative methods available for epilepsy research. Comparison table describing the characteristics of the alternative methods discussed in this review, including OSCs, iPSCs, brain organoids and zebrafish models. MoA, Mechanism of Action.

Multiple alternative methods have arisen in order to provide relevant insights into the epileptic pathology and accelerate treatment innovation (Figure 2). Among them, organotypic brain slice cultures (OSCs), Induced pluripotent Stem Cells (iPSC) and organoids appear as relevant models for new antiseizure drug candidates screening. On another hand, several models not classified as animals larval stage of *Danio rerio* (70) or non-vertebrates *C. elegans* (71, 72) and *Drosophila melanogaster* (73), traditionally used in basic research on embryonic development, have proven valuable in epilepsy research. This is because these models allow for high-throughput pharmacological screening, enabling the simultaneous evaluation of a large number of samples, the automated analysis of different phenotypes in short times, and the generation of avatars of human patients for the testing of new therapies. Among these, we will focus on the most widely used vertebrate zebrafish model.

### 3.1. *Ex vivo* and *in vitro* models

To identify new ASMs, it is key to employ a wide range of appropriate experimental approaches, including alternatives models. Thus, the establishment of these models/platforms ensures improved validity and relevance for their clinical use. Several alternatives to classical animal models based on *ex vivo* and *in vitro* models are currently available and being developed in the field of epilepsy.

#### 3.1.1. Organotypic brain slice cultures

Unlike conventional primary cell cultures, that allow the study of single cell populations, OSCs enable the simultaneous analysis of different cell types in a three-dimensional model, with preservation of some structural and synaptic organization features of the original tissue (74, 75).

In addition, OSCs allow the assessment of many aspects of relevance for the study of epilepsy and ASMs. Neurodegeneration, a possible consequence of seizures (76), can be evaluated through propidium iodide or other stainings, or even by measuring the levels of lactate dehydrogenase released to the medium (77). OSCs can be very useful to perform procedures that, although possible, are normally more challenging to carry out *in vivo*, including long-term live imaging (75), or electrophysiology (78). Recombinant adeno-associated viruses, commonly used to generate disease models *in vivo* (79, 80) can also be used to generate disease models in OSCs (78) and different compounds can be added into the culture medium to study them (81). As previously mentioned, they can support all the cell types found in the CNS, and therefore changes in cell types other than neurons, like glia and vascular cells, can also be studied.

Moreover, the use of OSCs significantly decreases the number of animal experiments that are considered severe, thereby promoting the principles of the 3Rs—reduce, refine, and replace (74).

However, many aspects of brain slice preparation can affect their viability and might influence neuronal connections. These aspects have been previously reviewed in detail by the ILAE (82). Briefly, the survival of the neurons depends on a variety of factors, including the species and age of the animal, the brain area selected, the medium composition and thinning of the slice. Nevertheless, contrary to what is observed in acute slices, where projection fibers are severed during the preparation, in OSCs the extended maintenance of the slices in an incubator with access to a cultured medium can produce a relatively stable cell viability, resulting in a long lifespan. Additionally, there is a significant synaptic rearrangement during the regrowth after slicing-induced deafferentation, but the properties of synaptic transmission are overall maintained (82).

Many brain areas have been used for OSCs, such as hippocampus, cortex, cerebellum and brainstem structures. In particular, organotypic hippocampal slice cultures have been broadly used to study epilepsy, because they allow for thorough and controlled investigation of the mechanisms behind epileptogenesis, while keeping the network phenotypic characteristics of epilepsy, especially the development of spontaneous seizures (83, 84). The most commonly used method for the preparation of organotypic hippocampal slice cultures was first described by Stoppini et al. (85) and later detailed by De Simoni and Yu (86).

Brain preparations derived from a variety of mammalian species, including rabbits, guinea pigs, rats, mice, and humans, have been shown to induce *in vitro* epileptiform activity (82). Most OSCs are generated from mice or rats before postnatal day 12, since at this developmental stage, the brain’s cytoarchitecture is well-established. Furthermore, the larger size of the brain at this stage makes it easier to handle, which allows neuronal cells to survive explantation. Additionally, explanted neuronal cells at this age exhibit greater plasticity, making them more resistant to the mechanical trauma that can occur when cutting neuronal processes (86).

Although most of the OSCs are generated from mice or rats, they have also been successfully established from tissue of adult patients. This represents a very good alternative to animal models since it allows to perform basic functional and mechanistic studies in a completely homologous model. Moreover, human OSCs preserve the complex neuronal cytoarchitecture and electrophysiological properties of human pyramidal neurons (87). However, it requires the availability of human tissue obtained from neurosurgery for refractory epilepsy (88). An example is the model of temporal lobe epilepsy in which the characteristic morphology and pathological activities are preserved, and epileptiform activities can be modulated by the addition of glutamatergic and GABAergic receptor antagonists (83).

#### 3.1.2. Induced pluripotent stem cells

iPSC technology has considerable potential for toxicity and efficacy drug screening and disease modeling, allowing the generation, growth, and study of human cells without the need for invasive isolation procedures or extensive ethical approval (89). Somatic cells obtained from patients can be reprogrammed to a pluripotent stem cell state which can then be differentiated into a broad range of different cell types, including neurons and glia (90, 91). iPSCs can be produced in about a month, and therefore, it is possible to rapidly generate a model with patient specific mutations with a lower cost than a mouse model. This is particularly relevant in epilepsy due to the heterogeneous nature of genetic epilepsies, with more than 500 loci listed as potentially causative when mutated and in some cases, such as in SCN1A-related epilepsies, over 1,250 distinct mutations identified in patients (92).

Several functional and molecular approaches can be used for the phenotyping of patient iPSC-derived neurons. Most of them are directed to study neuronal excitability, such as patch-clamp recording, which provides direct single cell measurements of electrical activity, multielectrode arrays (MEAs), for the measurement of electrical activity of a network of cultured neurons for extended periods, fluorescent assays of intracellular calcium or membrane voltage, and all-optical electrophysiology methods that allow high throughput studies. Other approaches for the phenotyping of iPSCs-derived neurons in the context of epilepsy include live cell imaging and omics studies (93).

Despite being a good model to study epilepsy, they also present some limitations. iPSC lines can have variable expression profiles and differentiation potential (92). In addition, it is very challenging to recapitulate the complexity of the brain and, despite the efforts to create brain circuits in 2D culture using iPSC-derived neural cells, the circuitry is still very different from the complex brain neuronal network. This is now improving thanks to the development of brain organoids made using 3D culturing technology (94).

Multiple patient-specific iPSCs derived disease models exist, generated from patient’s cells carrying specific mutations (95). These models have shown altered neuronal morphology, including soma size, neurite outgrowth, formation of synapse, and length of dendritic spine (92). The first *in vitro* model from a Dravet patient with a mutation in the SCN1A locus demonstrated how the primary cause of epileptogenesis seems to be the loss of function in GABAergic inhibition (96). After that, different studies have been published studying different mutations in the gene SCN1A (97, 98). Other diseases have also been successfully modeled using patient iPSCs, including Rett syndrome (99, 100) and Angelman syndrome (101) among others (92). These advancements have broadened the understanding of the disease etiology and pathology and set an extraordinary basis for the application of personalized medicine by developing targeted therapeutic strategies (102). In parallel to the development of iPSC, great advances in gene editing technologies have been made. This coincidence has considerably contributed to a fast expansion in the understanding of neurological disorders (103). CRISPR/Cas9 in iPSCs can be used to generate new models of various disorders, such as Alzheimer’s (104) and to generate isogenic pairs, which differ only by a single genetic modification, and are powerful tools to understand gene function. Furthermore, genome-wide CRISPR screens enable high-throughput investigation for genetic modifiers, opening up new pathways and revealing potential therapeutic targets (103).

CRISPR/Cas9 in human iPSCs was first used in epilepsy to generate a loss of function SCN1A mutation in order to gain more knowledge on DS. In this study, they fluorescently labeled GABAergic iPSC-derived neurons using CRISPR/Cas9 and studied their electrophysiology and the postsynaptic activity of inhibitory and excitatory neurons. They described a reduction in the amplitudes and an enhancement of the thresholds of action potential in patient-derived GABAergic neurons, together with a change in the postsynaptic activity from inhibitory to excitatory. These results further contributed to the previous knowledge on the physiological basis underlying epileptogenesis caused by SCN1A loss-of-function mutation (105). This strategy has been thereafter applied in several other studies, including more on the SCN1A gene (106), but also in other models of epilepsy, like in a model of KCNQ2 encephalopathy (95, 107). In this last study, they used patient iPSC-derived neurons and generated an isogenic mutation-corrected control line using CRISPR/Cas9, so that they could link phenotypic changes to the disease associated variant. They discover a functional enhancement of Ca^2+^- activated K^+^ channels, a rapid action potential repolarization and a larger post-burst afterhyperpolarization in the patient-derived neurons in comparison to the isogenic control ones. Once again, the combination of CRISPR/cas9 technology and iPSCs resulted in new findings that add to the previous knowledge on the disease mechanisms.

#### 3.1.3. Brain organoids

Brain organoids are organized structures composed of progenitor, neuronal, and glial cell types that closely resemble the architecture of the fetal human brain. Reprogrammed human iPSCs could undergo a self-organization process (108). To induce the formation of neural rosette structures, 3D aggregation of pluripotent stem cells, including both human iPSCs and ESCs, is facilitated in the presence of neural induction molecules, crucial step in the generation of brain organoids (109). Under optimal conditions, these cellular aggregates undergo self-organization, leading to the development of more complex and differentiated structures known as cerebral or brain organoids (110, 111).

Brain organoids replicate the human brain’s tissue structure and developmental pathway, in addition to its cellular composition, making them distinct from conventional two-dimensional (2D) cell cultures. As a result, they offer a unique opportunity to model human brain development and function, which may not be directly testable in direct experimentation (112). As with iPSCs, recent advances in genome editing, high-throughput single cell transcriptomics and epigenetics, have significantly advanced the use of brain organoids as a tool to study the development, evolution, and diseases of the human brain. This has resulted in a revolutionary expansion of our investigative capabilities (112).

In recent years, a novel approach has emerged as a second generation of brain organoids, known as brain assembloids, which offer a promising strategy for modeling human brain development and disease. Assembloids provide a solution by integrating multiple organoids or combining organoids with missing cell types or primary tissue explants (113). These assembloids use self-organization enabling complex cell–cell interactions, circuit formation, and maturation in long-term culture, distinguishing them from approaches that mix cell lineages in 2D cultures or use engineered microchips (114, 115). The successful growth and functional properties observed in assembloids composed of cortical, hippocampal, and thalamic organoids with active neuronal migration and interaction demonstrate the potential of these flexible, scalable, and controlled microfluidic systems for broad applications in neurological and biomedical research. It is anticipated that these innovative approaches will prove invaluable in unraveling human-specific aspects of neural circuit assembly and in modeling neurodevelopmental disorders using patient-derived cells. The integration of brain assembloids into the scientific landscape holds great promise for advancing our understanding of the human brain and developing targeted therapeutic strategies for neurological disorders as epilepsy (114).

Organoids have proven to be a valuable tool for exploring cellular phenotypes related to epilepsy. Nevertheless, the development of seizures and the replication of the electrophysiological properties of the brain in organoids, which are essential components of epilepsy research, are still active areas of investigation (116).

##### 3.1.3.1. Epilepsy progressive myoclonus 1

Di Matteo laboratory performed experiments using cerebral organoids derived from both Epilepsy Progressive Myoclonus 1 (EPM1) patients and healthy individuals (117). EPM1, an autosomal recessive disorder, is the most common form of progressive myoclonus epilepsy and associated with mutations in the cystatin B (*CSTB*) gene and its promoter. They found that *CSTB* overexpression in control organoids increases cell proliferation, whereas overexpression of a mutant form of *CSTB* led to its inhibition. Additionally, control organoids exposed to media from mutated organoids (from EPM1 patients) showed a decrease in cell proliferation, whereas media from control organoids rescued the proliferation deficit in EPM1 organoids. Low levels of functional *CSTB* result in an alteration of progenitor’s proliferation, premature differentiation, and changes in interneurons migration. This research manifested that the use of derived cerebral organoids provided valuable insights into the cellular and molecular mechanisms underlying this disorder.

##### 3.1.3.2. Developmental epileptic encephalopathies

Developmental epileptic encephalopathies are severe disorders characterized by intractable epileptic seizures and developmental delay where UDP-glucose-6-dehydrogenase (UGDH) gene has been implicated as a critical component, responsible for the conversion of UDP-glucose to UDP-glucuronic acid. Hengel et al. have recently generated cerebral organoids from patients with different mutations in the *UGDH* gene (118). Mutant organoids were significantly reduced in size and showed decreased expression of neuronal progenitor markers and proliferative cells. This study using cerebral organoids provides valuable insights into the molecular mechanisms underlying developmental epileptic encephalopathies and suggests potential therapeutic avenues, focusing on nutritional supplements and regulatory interventions. Remarkably, a similar experiment was performed with zebrafish, but *UGDH* mutant zebrafish did not show the same defects, indicating different responses between the organoid and zebrafish models. This fact underscores the importance of studying different models of the disease to gain comprehensive insights, as each model contributes unique aspects to our understanding and contributes to a more holistic understanding of the disease.

##### 3.1.3.3. Additional disorders

Recently some advances in this field have been made, with the successful establishment of brain organoid models of Angelman syndrome showing among other features hyperactive neuronal firing (119), and Rett syndrome, with susceptibility to hyperexcitability and recurring epileptiform spikes. This last model was also used to test valproic acid (VPA) and the TP53 inhibitor pifithrin-α(PFT) as possible treatments for this syndrome (120). Furthermore, another study succeeded in the development of a brain organoid model of developmental and epileptic encephalopathies (DEE), demonstrating not only the presence of epileptiform activity, but also showing the utility of this model for the molecular study of epilepsy (121). Although more studies are needed to enhance the accuracy of these disease models, they are promising tools for the evaluation of future treatments in epilepsy.

##### 3.1.3.4. Therapeutics testing with brain organoids

Brain organoids provide a unique and valuable platform for gaining insight into complex neurological diseases. However, the current state of organoids is characterized by their simplicity and as a consequence of being *in vitro* models, the knowledge derived from them may carry intrinsic limitations. While brain organoids are valuable models, they have certain limitations in recapitulating the complex tissue structure and functions of the human brain, particularly with respect to the choroid plexus (ChP). The ChP plays an important role in cerebrospinal fluid (CSF) secretion and the formation of the blood-CSF barrier. To overcome this limitation, researchers have made efforts to establish human ChP organoids capable of simulating selective barrier properties and CSF-like fluid secretion within self-contained compartments. An exciting feature of these ChP-CSF organoids is that they exhibit similar small molecule selectivity as observed *in vivo* (122). This property makes them valuable tools for predicting the CNS permeability of new compounds. Given the growing demand for more effective CNS drugs, it is critical to avoid the shortcomings of drug candidates that enter clinical trials only to fail due to lack of efficacy, limited CNS penetration, or translatability issues from animal models.

Further technological development is required to advance the field and increase the utility of brain organoids as reliable models. Efforts toward accelerating functional maturation to more closely resemble the *in vivo* state, as well as incorporating additional cell and tissue types, should be directed toward creating more comprehensive and faithful representations of the human brain. These advances will contribute significantly to the reliability and relevance of brain organoids in the study of neurological disorders (112).

### 3.2. The zebrafish model

While the zebrafish model has been extensively used in classic developmental studies for many years, in particular in neurodevelopment (123), in the last two decades it is being exploited for target validation and drug screening (124, 125).

Zebrafish provides a large variety of possibilities in order to explore the underlying principles of seizure generation in multiple epilepsy models (126). With their small size, high breeding rate, rapid development and relatively low maintenance costs, in addition to their ability to take up compounds from the water surrounding them, zebrafish larvae are particularly suited to perform high-throughput phenotype-based drug screening (127). In addition, zebrafish exhibit genetic similarities with humans and present numerous advantages for genetic manipulation. Advanced and efficient genome manipulation techniques have facilitated the creation of models for various genetic epilepsies and disorders where seizures are a primary symptom (58, 127). Moreover, zebrafish larvae possess analogous brain structures to those present in mammals and exhibit a diverse range of complex behaviors, which can be susceptible to seizures, within just a few days post-fertilization (128).

Cortical and subcortical structures of zebrafish larvae are conserved and maintained in relation to their characteristic cellular features and main connections. The main sections in which zebrafish brain is subdivided include forebrain, midbrain and hindbrain/spinal cord. During early development, further subdivisions occur, giving rise to specialized structures in the adult brain which can also be found in rodent models and humans: pallium, subpallium, thalamus, and cerebellum (70). Moreover, some structures are highly homologous between humans and zebrafish, including the habenula (129), striatum, basal ganglia (130, 131), and cerebellum (132).

The similarities between zebrafish and mammalian (human and rodent) models are remarkable, both in terms of general brain organization and cellular morphology (128). In particular, the zebrafish amygdala and habenula are involved in affection-related behaviors, mirroring human data on these brain structures. The habenula, a group of nuclei in the epithalamus, plays a role in regulating the release of serotonin and dopamine (133), making it an experimentally feasible system for dissecting vertebrate brain circuits (134). This conservation allows for the study of brain substrates in zebrafish and their translational value for the study of pathological behavior, as habenular hyperactivity has been observed in humans with depression and in rodent models of this disorder (135).

In terms of brain neurochemistry, zebrafish share a highly conserved profile with humans and rodents. They possess all major neuromediator systems, including neurotransmitter receptors, transporters, and enzymes involved in synthesis and metabolism (136–138).

Zebrafish also have well-developed functional neuroendocrine systems, analogous to those found in mammals. The neuroendocrine system remains conserved in zebrafish (ZF) and for hypothalamus development, the same genes as in mammals are employed. Additionally, the majority of neuropeptidergic systems and neurotransmitters exist in this model (139, 140). Stress responses in zebrafish, similar to humans, are mediated by cortisol, which is activated by hypothalamic–pituitary hormones and acts through glucocorticoid receptors (141, 142). Zebrafish cortisol responses closely resemble behavioral indicators of stress and can be genetically and pharmacologically modulated (141–143). These similarities make zebrafish a valuable model for studying CNS disorders.

In order to study epilepsy, zebrafish allow the performance of multiple bioassays. Notable advantages include the capacity to perform *in vivo* brain imaging through activity-dependent fluorescent/bioluminescent reporters, EEG recordings in both larval and adult fish, and high-throughput behavioral analysis by means of automated video tracking systems (58, 144). Regarding seizure evaluation, zebrafish has the ability to mimic motor behaviors observed in humans, including changes in swimming patterns and body shaking (58).

Overall, taking into account all these characteristics, the zebrafish model is suitable for investigating the source of these disorders as well as the series of events leading to their onset. Additionally, it serves as a high-throughput *in vivo* drug screening platform for compounds with anti-seizure potential (58).

#### 3.2.1. Pharmacological models (PTZ and kainic acid)

##### 3.2.1.1. Pentylenetetrazole model

The PTZ model was first described in zebrafish in the early 2000 (145). Consecutive studies then concluded that zebrafish larvae at 7 days post fertilization (dpf) exhibit electrophysiological, behavioral and molecular changes similar to the rodent PTZ models (58, 146). In rodents, the dose of PTZ required to induce seizures may vary depending on factors such as strain, sex, age, and route of administration (primarily intraperitoneal injection). PTZ is primarily used as a screening tool for ASDs in rodents rather than to study the pathophysiology of epilepsy. Different types of seizures are reproduced at different doses of PTZ, with low doses inducing absence seizures and higher doses inducing generalized tonic–clonic seizures. Commonly used protocols for PTZ administration in mice during antiseizure drug screening aim to induce clonic seizures lasting at least 5 s in at least 97% of animals within 30 min (147, 148). Similar to rodents, PTZ in the ZF is considered a model for generalized seizures, particularly absence and generalized tonic–clonic seizures.

Zebrafish larvae are capable of eliciting seizure-like behavior when immersed in a volume containing PTZ. This compound is absorbed by the gills, gut or skin and eventually reaches the brain (58). Within seconds or minutes, switches in locomotor activity are detected. These movements are characterized by a series of events, starting from Stage I, consisting of accelerated movements around the periphery of the behavioral chamber. During stage 2, ZF larvae perform “whirlpool-like” movements. The epileptic behavior concludes with stage 3, which takes place in case of high PTZ concentrations. ZF larvae experience loss of posture, rapid and uncontrolled movements, intermittent pauses and occasional stiffening of the body (145). The locomotor behavior induced by PTZ displays a correlation with the electrical activity of the brain determined by EEG. This behavior is characterized by spontaneous epileptiform discharges, which manifest variations in frequency, amplitude and duration depending on the timing of PTZ exposure (145).

This model has enabled the standardization of simple locomotion assessments (tracked using software analysis) and electrophysiological tests for quantifying and monitoring seizures in zebrafish larvae (146). Subsequently, the zebrafish PTZ model has gained popularity in laboratories worldwide and has demonstrated consistency with the rodent PTZ models in validating antiepileptic drug candidates. This emphasizes the importance of zebrafish as a fast and robust model for ASMs screening (149).

Multiple ASMs with known effect in the rodent PTZ model, have been tested in zebrafish. During the study performed by Gupta et al., ZF were exposed for 15 min to a 6 mM PTZ solution co incubated with standard ASMs in order to monitor their anti-seizure activity (150). These compounds were valproic acid, carbamazepine, diazepam, gabapentin, carbamazepine, pregabalin and lacosamide. Lacosamide, valproic acid, gabapentin, carbamazepine and diazepam presented a concentration dependent increase in latency during all stages of seizures. For lacosamide it was significant at 100 μM to 3 mM, for valproic acid at 300 μM to 10 mM, for gabapentin at 1–10 mM, carbamazepine at 10–100 μM and diazepam 30–100 μM (150). Pregabalin by contrast, did not increase seizure latency compared to the vehicle control (PTZ 6 mM).

Efficacy data of ASMs obtained from the zebrafish model compares to the rodent one. Carbamazepine at 20 mg/kg, sodium valproate at 300 mg/kg, diazepam at 1 mg/kg were tested in this rodent model and showed protection from clonic seizures (151). Pregabalin tested at 200 mg/kg did not cause a significant reduction of clonic seizures compared to the vehicle control, as described in the PTZ zebrafish model (150).

##### 3.2.1.2. Kainic acid, novel model by pericardial injection

Kainic acid (KA) is defined as a potent agonist of AMPA/KA glutamatergic receptors. It induces network reorganization, excitotoxicity and neuronal death in different brain regions. Since it produces acute seizures in rodents through systemic injections and recurrent seizures mimicking a chronic model of temporal lobe epilepsy by intracerebral injections, it is a widely utilized proconvulsant drug (48, 58).

KA is considered a model in adult ZF that is reported to reproduce seizures, similar to its use in rodents (152). In ZF, the majority of KA studies have been conducted in adult animals. In these studies, KA is administered intraperitoneally to induce seizure-like behavior, resulting in clonic convulsions observed in all ZF treated at a dose of 6–8 mg/kg (152). It is noteworthy that these doses are comparable to those commonly used in rodent models (6–15 mg/kg) (153).

Previous efforts in order to trigger seizures in zebrafish larvae by incubating them in KA solution failed to produce the desired seizure phenotype. According to the study performed by Kim et al. (154), KA perfusion by means of artificial cerebrospinal fluid immediately led to local electrographic brain discharges. Additionally, Alfaro et al. (152) observed that adult zebrafish intraperitoneally injected with KA presented convulsions mimicking clonus. The results of these studies imply that the high hydrophilicity of KA prevents ZF larvae from efficiently absorbing it when dissolved in tank water (155).

In 2021, a novel KA model was introduced in zebrafish larvae (155). This KA-induced zebrafish epilepsy model is achieved by intrapericardial injection of KA in 3dpf zebrafish larvae. Due to a shift in balance between GABAergic inhibition and glutamatergic excitation, larvae show whole brain abnormalities and involuntary seizure-like movement patterns shortly after injection. After the latency phase, larvae also experience epileptiform brain discharges (155). Following treatment with commonly used ASMs, as topiramate 100 μM, tiagabine 100 μM and carbamazepine 100 μM, a reduction in epileptiform discharges was observed while none of the compounds tested decreased seizure-like behavior (155). Multiple ASMs were also tested in the kainate mouse model of mesial temporal lobe epilepsy obtained by unilateral injection of kainate into the dorsal hippocampus (156). All the compounds tested: valporate (300 mg/kg), lamotrigine (90 mg/kg), carbamazepine (75 mg/kg), levetiracetam (600 mg/kg), pregabalin (50 mg/kg), phenobarbital (20 mg/kg), diazepam (1 mg/kg), tiagabine (0.3 mg/kg), and vigabatrin (50 mg/kg) acutely reduced the occurrence of hippocampal paroxysmal discharges (156).

The kainic model described above, provides useful insights into the mechanisms of seizures and epileptogenic processes and could possibly be applicable in the future for the discovery of novel therapeutics including disease-modifying strategies in the fight against drug-resistant epilepsies (155).

#### 3.2.2. Genetic models

Another common approach for epilepsy studies is based on the modulation of epilepsy-associated genes. The rodent brain has a long maturation time, which makes it challenging to determine the optimal timing for pharmaceutical intervention in epilepsy studies, even with various rodent genetic models available. In contrast, using zebrafish epilepsy models could be more useful in researching the epileptogenic pathway related to genetic abnormalities. Also, since most genetic epilepsy syndromes occur in childhood, studying larval zebrafish can be an effective method to monitor brain development.

Given the rather recent inclusion of the zebrafish in epilepsy research, in most cases the widespread antisense morpholino strategy has been used for disease in early days. Through this methodology the knockdown of several genes such as *kcnj10* (157, 158), *kcnq3* (159), *stx1b* (160), *chd2* (16, 161) has been reported to induce severe behavioral alterations (epileptic discharges, poly-spikes, paroxysmal discharges). Nevertheless, given the variable results that might be obtained comparing studies in mutants and morphants (162) mostly due to genetic compensation mechanisms induced by loss-of-function mutations and mutant mRNA degradation (163, 164) the gold standard model for zebrafish epilepsy research is a mutant line carrying a loss-of-function mutation in domain III of the voltage-gated sodium channel *scn1Lab* (165). The zebrafish gene, *scn1Lab*, is highly homologous to the human gene SCN1A, with 77% of DNA identity. In the developing zebrafish brain, *scn1Lab* is expressed widely, especially in the forebrain, optic tectum, and cerebellum. Frameshift or missense mutation in this gene can lead to the onset of DS, a severe form of genetic pediatric epilepsy that causes developmental disabilities and persistent drug-resistant seizures. *scn1Lab* gene disruption in zebrafish is able to recapitulate human epileptic phenotypes. Specifically, zebrafish with a mutated *scn1Lab* gene show spontaneous seizures detected through electrophysiological recordings, similar to epilepsy in humans. When challenged with a light dark (LD) transition assay, mutant zebrafish exhibit abnormal locomotor patterns, with consistently higher activity levels (hyperlocomotion) compared to their wild-type siblings. In the pioneer study where the mutant was characterized (165), the model was challenged with over 300 compounds in a phenotype-based screening. As a result, Clemizole (EPX-100), an FDA-approved compound with anti-histaminic properties, was found to be effective in inhibiting seizures in the mutant fish and has passed through phase I clinical trials as an “add-on treatment” for DS. Starting from this success case, other drug repurposing screening have been conducted using the *scn1Lab* mutant and identified several drugs like fenfluramine (144) (now FDA-approved as Fintepla^®^), synthetic cannabinoids (166) (similar to the FDA-approved cannabidiol Epidiolex^®^), trazodone (Desyrel^®^), and lorcaserin (Belviq^®^) (167), which have also shown promise in treating DS in zebrafish experiments. These findings demonstrate how quickly discoveries in zebrafish can lead to potential clinical treatments for DS.

Although the aforementioned repurposing studies are based on the use of the same genetic mutant background, the advent of CRISPR/Cas9 and the continuous refinement of the technologies based on this system offer now the possibility of inducing mutations with high efficiency in human epilepsy-associated genes. Along this line, in a recent study (168) a range of loss-of-function single gene mutations identified through genome wide association (GWAS) represented the starting point for the generation of 37 mutant zebrafish lines carrying deletions in the selected loci. Among these, 8 lines (homozygous mutant for *arxa*, *eef1a2*, *gabrb3*, *pnpo*, *scn1lab*, *strada*, and *stxbp1b* and heterozygous for *grin1b*) result in recurrent electrographic seizures, thus opening new avenues for the study of the pathophysiology of rare disease and at the same time expanding the portfolio of lines that can be used for high-throughput screenings of ASMs.

Despite the fact that the generation of isogenic lines is crucial for the assessment of loss of function phenotypes, the time required for the obtention of mutant lines, including husbandry of fish, crossing for two generations and genotyping does not meet the current needs of personalized therapy based on genetic background of the affected individuals. Indeed, new genetic targets and genomic variants involved in epilepsy pathophysiology are being identified quickly through large-scale exome sequencing studies of cohorts of patients. This requires the development of high-throughput methods for timely generation of animal disease models to test the efficacy of compounds modulating these targets. To reduce the generation time for genetic target validation and the characterization of loss-of-function alleles in zebrafish, a variety of CRISPR/Cas9-based methods have been improved. The continuous refinement of single guide RNA (sgRNA) and Cas9 synthesis for the targeting of genes of interest has reached such efficiency that it is possible to induce gene loss-of-function already in the F0 generation. This is achieved by induction of high rates of open reading frame disruption mutations in microinjected zebrafish embryos, which are somatic mutants or CRISPANTs (169, 170). This transient approach makes it possible to directly identify and analyze mutant phenotypes and shortens the time and expense needed to achieve homozygosis in the F2 generation.

CRISPANTs models have been generated for human indications (9), epilepsy being one of them. Indeed, in a recent report (171), the behavioral fingerprint, intended as multiparametric analysis of larval behavior derived by tracking the animals over time, of *scn1lab* zebrafish homozygous mutant and F0 CRISPANTs for the same gene have been compared. Interestingly, the F0 knockouts phenotypes highly correlated with the mutant phenotype, being the behavioral fingerprint of both groups significantly different from their wildtype counterpart.

The use of CRISPANTs could be crucial for the high-throughput generation of novel zebrafish epilepsy mutants and allow antiepileptic drug screening already in F0 larvae, enabling fast-track personalized treatment design.

At the same time, a wide array of strategies has been developed, in order to precisely insert human mutations into the zebrafish genome. The gold standard technique for precise gene modification is based on Homologous directed repair (HDR), which involves the use of template DNA carrying the desired sequence change to substitute the sequence at the target locus following a double-strand break (DSB) by the CRISPR/Cas9 system (172). HDR-genome editing, however, is linked to significant amounts of off-target mutations and insertions/deletions byproducts. To overcome these issues, base editing, which uses a DNA C or A deaminase enzyme coupled to the Cas9 nickase protein to install precise modifications without the need for donor DNA or DSBs (173), was firstly developed and it has shown a great efficiency even in F0 in zebrafish larvae (174). Finally, a key breakthrough in the field of genome editing is the Prime editing (175). This technique is based on the fusion of a Cas9 nickase to a Reverse Transcriptase. In this case, the sequence of interest is copied into the target locus by reverse transcription of an RNA template sequence, thus avoiding double strand break and drastically reducing unintended DNA mutations at the target locus. The implementation of Prime editor proteins in zebrafish has led to promising results, with relatively high percentages (up to 30%) of correct edits in F0 embryos (176).

All these strategies are being employed at a fast pace and already allowing the development of humanized zebrafish in a short time frame, thus paving the ground for future customized high-throughput drug screenings.

## 4. Discussion and future perspectives on the use of alternative models

More than 20 years have passed since the signing of the Bologna declaration in 1999, at the third World Congress on Alternatives to the Use of Animals in the Life Sciences. The proclamation established the requirement to abolish cruelty in science before it could be applied to humans, encouraging the strict implementation of the 3Rs (replace, reduce, and refine) in processes involving laboratory animals. Since then, there have been many changes that have occurred in the regulation of animal experimentation and advances in the search for and validation of alternative models.

Here, we have presented some of the alternatives to current methods applied to epilepsy research. Although the classic models greatly contributed to the development of multiple drugs to treat epilepsy, there is still a high percentage of patients with no seizure control, partly due to the development of drug resistance, but also to the lack of accurate models to study the mechanisms underlying epileptogenesis. The continuous advances in the development of NAMs (new approach methodologies) has the potential to fill this gap in epilepsy research, while contributing to the implementation of the 3Rs. Moreover, the success in the use of Clemizole in a zebrafish model of DS and other drug repurposing screenings (144, 165, 166), has proved the benefit of the use of novel model to translate the results into potential clinical treatments.

Nevertheless, a consistent change in animal experimentation pushing forward the 3R principle in neurological disorders and other human indications can be achieved only with a strong coordinated effort led by governmental agencies, international institutions, pharmaceutical and chemical industry, academia and animal welfare organizations.

### 4.1. Advancements in regulation of use of NAMs in research

Importantly, the use of NAMs such as *in vitro* and non-animal models, some of which we have presented in this overview, is gradually gaining momentum in novel policies adopted by regulatory agencies worldwide. For example, since 2011, the European Medicines Agency (EMA) has supported Directive 2010/63/EU in a number of ways (177). One of them is the establishment of the “3Rs Working Party” (3RsWP), which encourages the adoption of alternative techniques and supports drug developers who are dedicated to minimizing the use of animals during the regulatory process. Organization for Economic Cooperation and Development (OECD) guidelines have been established to assist businesses in creating alternate techniques for determining if chemicals are safe enough to register with the European Chemicals Agency (ECHA). In the USA, the FDA (Food and Drug Administration)'s NCTR (National Center for Toxicological Research) (178) division works to develop and validate alternative (*in vitro* and *in silico*) toxicity evaluation techniques. The last step forward on this matter is the FDA Modernization 2.0 Act (179), signed by Joe Biden, president of the United States, at the end of 2022. This mandate is groundbreaking since it ends a 1938 federal mandate according to which experimental drugs had to be tested on animals before being used in human clinical trials. Today, the alternative methods accepted by U.S. agencies to reduce or replace experimental animals is as high as 128 (180).

### 4.2. A combinatorial approach for discovery and testing of new ASMs

All these initiatives that suggest an important change in global drug discovery pipelines not restricted to the epilepsy field, raise the questions of how NAMs can eventually completely replace animal experimentation, providing safe treatments for patients in a more ethical and sustainable manner. Here, we have extensively reviewed alternative models for the discovery of novel therapeutics in epilepsy, with their relative advantages and limitations and we do believe that the answer to the aforementioned question relies on a comprehensive approach that integrates data from different methods. A relatively novel concept in toxicity assessment of chemicals for regulatory purposes is based on the IATA, Integrated approaches for testing and assessment (181). IATA rely on the combination of a variety of information sources to infer hazard for chemical risk assessment. A similar strategy could be used to evaluate the potential efficacy of novel ASM compounds. Following a IATA framework, the first step would be to collect all available information through a literature review on generated data about the compound of interest, if a repurposing approach is used, or the chemical class, in the case of a newly synthesized molecule. Additional testing using the multiple models presented would help inform on the effect of a compound at different levels of complexity (e.g., molecule, cell, organ, tissue, organism). The individual outcomes deriving from the presented *in vitro*, *ex vivo* or whole organism would be integrated and decision frameworks can be established for the analyzed chemicals. If results are concordant in orthogonal assays with NAMs, the compounds would progress to physiologically based kinetic (PBK) modeling for *In-Vitro*-to-*In-Vivo*-Extrapolation (IVIVE) (182, 183). IVIVE uses physiologically based kinetic (PBK) models to estimate a human equivalent dose that can be compared with estimated human exposures (reverse dosimetry) or estimate internal doses (blood, tissue levels) based on a specified exposure for comparison with *in vitro* bioactive concentrations (forward dosimetry). In this case there would be no need for further animal experiments. When discordant results are obtained, additional tests with NAMs or rodent models, in this case in a much reduced number since extensive information has been generated with previous steps, might be required to take a decision on the tested chemical.

Overall, applying an integrated strategy with data proceeding from multiple sources would greatly reduce and eventually replace animal testing.

It could be expected that the integration of results obtained with experiments in NAMs coupled with the advancements in high-throughput disease modeling via genome editing will enable development of personalized treatment approaches not only in epilepsy but also for other human indications.

Throughout the review, we have mentioned a few strategies to tackle the genetic variability underlying phenotypic heterogeneity of epilepsy among which stable and somatic Knockout generation for loss-of-function alleles, base editing and prime editing for accurate insertion of single nucleotide polymorphisms (SNPs). These methodologies can be either used for disease modeling or for disease-associated mutation corrections for SNPs or even more complex scenarios as repeated expansions. For example, a recent study reported successful excision of hexanucleotide repeat expansions in patient-derived iPSC neurons, brain organoid and mouse models of ALS amyotrophic lateral sclerosis (ALS) and frontotemporal dementia (FTD) (184). These tools can be virtually applied in any model of interest for a selected indication, broadening the possibilities to discover novel therapeutics.

To conclude, all these initiatives confirm that we are in a change of era in biomedical research and drug discovery. Over the next 5 years, it is likely that the use of cell based models or larval models as zebrafish will continue to grow in research as scientists seek to reduce reliance on traditional animal models and develop more efficient and ethical methods of disease modeling, drug discovery and toxicology testing.

## Author contributions

CM, MM, DC, GS-E, and VD conceptualized the study and reviewed and edited the manuscript. CM and MM performed bibliographic research. DC conceptualized and prepared the supporting figures. GS-E and VD prepared the original draft. All authors have read and agreed to the published version of the manuscript.

## Conflict of interest

All authors are employees of ZeClinics SL, a contract research organization using the zebrafish model for research in disease modeling, target validation and drug screening.

## Publisher’s note

All claims expressed in this article are solely those of the authors and do not necessarily represent those of their affiliated organizations, or those of the publisher, the editors and the reviewers. Any product that may be evaluated in this article, or claim that may be made by its manufacturer, is not guaranteed or endorsed by the publisher.
